# Editorial: Acknowledging Those Who Did the Work

**DOI:** 10.1523/ENEURO.0490-18.2018

**Published:** 2018-12-21

**Authors:** Christophe Bernard

**Affiliations:** Editor-in-Chief

Dear fellow scientists,

Many research studies require a lot of time, often spanning several years, in particular when several rounds of revisions are needed. In many instances, several of the original contributors have left the lab before the story is finally accepted. Many of us give small projects to rotating students, which may end up as a full figure, a panel in a figure, or supporting information. In the end, many individuals may have contributed to the story. Although the rotating students, PhD students, and postdocs are usually acknowledged as authors, their contribution is often diluted by the fact that their names appear in the middle of the author listing, between the first and last authors. Most scientific journals include a list of contributions (A, B, and C performed the experiments; A, C, D, and F analyzed the data, etc.), but it is not exactly clear who did what. Therefore, I believe that it is important for each contributing author to be able to show precisely what they have done. This may be important for a CV, a letter of motivation, etc., to join another lab, for example.

*eNeuro* is proud to introduce a new initiative to precisely acknowledge everyone who is directly responsible for the work. The corresponding authors will now be offered (at submission or before publication) the possibility to include at the end of each figure legend the contributions of each individual to data acquisition and analysis. Thus, it will be clear to assess who did what. I think this is only fair to all people who contributed. To build a house, every brick is important; it is time to acknowledge who provided which bricks.

